# The protective effects of enriched citrulline fermented milk with *Lactobacillus helveticus* on the intestinal epithelium integrity against *Escherichia coli* infection

**DOI:** 10.1038/s41598-020-57478-w

**Published:** 2020-01-16

**Authors:** Sze Wing Ho, Hani El-Nezami, Nagendra P. Shah

**Affiliations:** Food and Nutritional Science, School of Biological Sciences, The University of Hong Kong, Pokfulam Road, Pokfulam, Hong Kong

**Keywords:** Microbiology, Nutrition

## Abstract

This study examined the protective effects of citrulline enriched-fermented milk with live *Lactobacillus helveticus* ASCC 511 (LH511) on intestinal epithelial barrier function and inflammatory response in IPEC-J2 cells caused by pathogenic *Escherichia coli*. Five percent (v/v) of fermented milk with live LH511 and 4 mM citrulline (5%LHFM_Cit-4mM) significantly stimulated the population of IPEC-J2 cells by 36% as determined by MTT assay. Adhesion level of LH511 was significantly increased by 9.2% when incubated with 5%LHFM_Cit-4mM and 5%LHFM_Cit-4mM reduced the adhesion of enterohemorrhagic (EHEC) and entero-invasive (EIEC) *E. coli* in IPEC-J2 cells by 35.79% and 42.74%, respectively. Treatment with 5%LHFM_Cit-4mM ameliorated lipopolysaccharide (LPS) from *E. coli* O55:B5 induced activated inflammatory cytokines expression (TNF-α, IL-6 and IL-8) and concentration (IL-6 and IL-8) and early apoptosis. It restored the transepithelial electrical resistance (TEER) and regulated the expression and distribution of tight junction (TJ) proteins (zonula occluden-1 (ZO-1), occludin and claudin-1), toll-like receptors (TLRs) (TLR2 and TLR4) and negative regulators of TLRs signalling pathway (A20 and IRAK-M). In conclusion, our findings suggested that 5%LHFM_Cit-4mM might have the positive effects on improving and maintaining the intestinal epithelial cell integrity and inflammatory response under both normal and pathogenic LPS-stimulated conditions.

## Introduction

Probiotics are defined as live micro-organisms that contribute health-promoting effects^1^. These organisms have been extensively investigated for their health-promoting effects on intestinal tract and modulating the intestinal epithelial barrier functions by several mechanisms, such as prevention against pathogenic adhesion, reinforcement of tight junctions (TJ), protective effects against damage on TJ, suppression of inflammatory cytokines and anti-apoptotic effects^2^.

Pathogenic infection occurs when pathogen adheres to host intestinal epithelium^3^. The infection of pathogenic *Escherichia coli* strains, such as enterohemorrhagic (EHEC) and enteroinvasive (EIEC) *E. coli*, begins with the colonization and adherence to the intestinal mucosal surface and thus causing damage to the host^4^. The ability of probiotics to adhere to the intestinal epithelial cells is considered as a possible mechanism contributing to the inhibition effect against pathogenic adhesion by competing the adhesion site or/and for nutrients^3^ or producing antimicrobial agents^5^. Several *Lactobacillus* strains, such as *L. rhamnosus GG*, *L. fermentum*, *L. acidophilus* and *L. plantarum*, have been demonstrated to adhere to the intestinal mucus and markedly diminish the adhesion of *Bacteroides vulgatus*, *Clostridium histolyticum*, *Enterobacter aerogenes* and *Staphylococcus aureus*^3^.

Impaired intestinal barrier functions following pathogenic infection include TJ dysfunction, including increased epithelial permeability and damages to epithelial structure, trigger of pro-inflammatory response and excessive apoptosis. With regard to the importance of TJ integrity on intestinal barrier function, the role of TJ is to establish a selective paracellular pathway by sealing the apical epithelium and endothelium to control the transport of solutes^6^. TJs consist of various types of protein, including peripheral membrane proteins and transmembrane proteins. The major components of TJs are zonula occludens 1 (ZO-1), which are members of peripheral membrane proteins, and occludin and claudin-1, which are members of transmembrane proteins^7^. Some probiotics have been proven to enhance TJs integrity and prevent alterations of TJs due to pathogenic damage by stimulating TJs protein expression and distribution^8,9^. For instance, treatment with *L. plantarum* improved TJs functions in both normal and pathogen infected conditions; it also induced expression level of occludin and ZO-1 in normal condition *in vitro*^10^ and increased localization of these proteins in the TJs in healthy subjects^11^. Furthermore, it also ameliorated the epithelial permeability by restoring the transepithelial electrical resistance (TEER) level in pathogenic lipopolysaccharides (LPS)-infection *in vitro*^12^.

The positive effects of probiotics in modification on intestinal barrier have also been linked to their ability to regulate toll-like receptors (TLRs) and negative regulators^2^. Activation of TLR2 and TLR9 and suppression of TLR4 are considered as anti-inflammatory^13^. A previous study reported that *L. rhamnosus* GG (LGG) stimulated TLR2 expression accompanied by diminished IL-6 level^14^. Excessive inflammation is associated with the high expression of TLR4, thereby abnormal stimulation of TLR2 and TLR4 has been found in ulcerative colitis model. Similarly, the application of probiotics treatment has been shown to regulate the impaired expression of TLRs and therefore attenuated the inflammatory responses^15^. Modulation of TLR2 of probiotics regulated the pro-inflammatory response via increasing the production of several negative regulators to suppress TLR4 expression^16^. Shimazu, *et al*.^17^ also found that *L. jensenii* regulated the expression of IL-6 and IL-8 induced by entertoxigenic *E. coli* and LPS through stimulating negative regulators (A20, Bcl-3 and MKP-1).

Probiotic-fermented milks have been widely used as the commercial products for promoting health-benefits and therapeutic effects, including their role in relieving the symptoms of lactose intolerance and diarrhea and decreasing blood pressure^18^. However, there is limited information on the effects of probiotic-fermented milk on the intestinal epithelium. An early study by Thoreux, *et al*.^19^ reported that *L. paracasei* DN114001 fermented milk supernatant showed the growth promoting effect via enhancing proliferation of IEC-6 cells. Recently, Chen, *et al*.^20^ also determined the cell growth promoting and epithelial integrity strengthening effects of *L. paracasei* 01 fermented milk supernatant on Caco-2 cells. *L. helveticus* is a common *Lactobacillus* strain for making fermented products, koumiss and beverages^21^. Milk fermented by *L. helveticus* has been reported to alleviate hypertension via producing the functional peptides, Val-Pro-Pro (VPP) and Ile-Pro-Pro (IPP); these peptides show the inhibition of angiotensin-converting enzyme (ACE)^22^. Apart from the anti-hypertension effect, *L. helveticus* isolated from fermented products is also reported to improve calcium absorption^21^, enhance the immunological defences^23^, attenuate pro-inflammatory response and increase the production of anti-inflammatory cytokines^24^.

Citrulline is a non-protein amino acid, abundant in watermelon and can be generated from arginine^25^. Citrulline has been proven to be utilized by some *Lactobacillus* strains to generate ATP through arginine deaminase (ADI) pathway for cell growth^26^. Moreover, owing to the ability of citrulline to re-produce arginine via argininosccinate lyase (ASL) and argininosuccinate synthetase (ASS)^27^, it has been suggested to increase the bioavailability^28^ and exert the same advantageous effects of arginine^25^. Supplementation of citrulline has also been investigated on its contribution to beneficial effects on the intestinal tract, such as maintaining TEER in hypoxia-induced injury *in vitro*^29^, stimulating protein synthesis and promoting repair after injury in *in vivo*^27,28^.

Therefore, in this study, we hypothesized that citrulline-enriched fermented milk with live *L. helveticu*s may be a novel functional supplement for enhancing the intestinal barrier function and ameliorating the adverse effects of pathogens. Thus, the aim of this study is to investigate the effects of citrulline enriched fermented milk with *L. helveticu*s on IPEC-J2 cells infected by *Escherichia coli*/treated with lipopolysaccharides (LPS) on the regulation of inflammatory response and TJ integrity.

## Results

### Effects of fermented milk, citrulline and *Lactobacillus helveticus* ASCC 511 on IPEC-J2 cell growth

**Figure 1:**
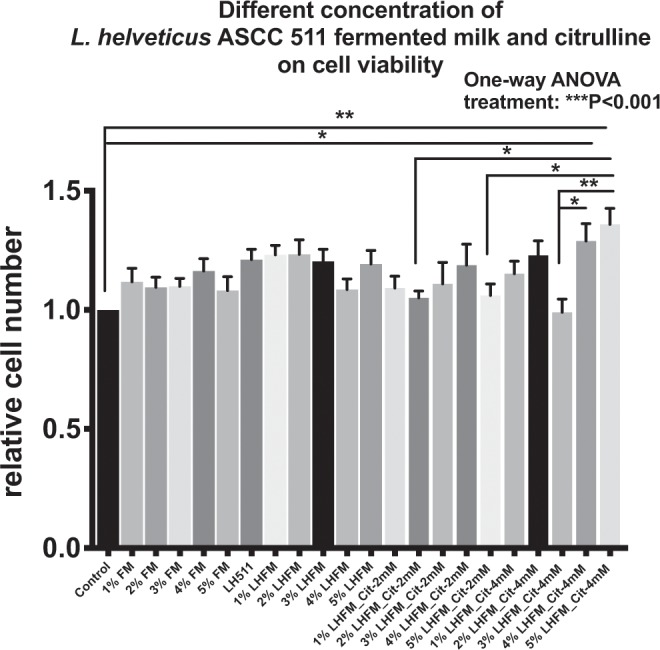
Effects of different concentration of *L. helveticus* ASCC 511 fermented milk and citrulline on cell viability of IPEC-J2 cell by MTT assay. Results are represented as mean ± SEM, n = 8. Significance shown for the difference compared between treatments as ***P < 0.001 by One-way ANOVA and *P < 0.05 and **P < 0.001 as the difference compared between groups by Tukey’s multiple comparisons test.

The cell viability of IPEC-J2 cells in different concentration of LH511-removed fermented milk supernatant (FM), LH511 alone, FM with live 3 × 10^7^ CFU/ml LH511 (LHFM) and LHFM with citrulline was determined by MTT assay. Figure 1 shows that FM, LH511 alone, LHFM and LHFM with citrulline were not cytotoxic to IPEC-J2 cells (P < 0.001). It shows that 1–5% (v/v) FM, LH511 alone and 1–5% (v/v) LHFM increased the relative cell number compared with control, although they were not statistically significant (all P > 0.05). It suggests that 4%LHFM_Cit-4mM and 5%LHFM_Cit-4mM significantly improved the population of IPEC-J2 cells with 29% and 36% increase compared with the control (both P < 0.05). Dose-dependent effects on the growth of IPEC-J2 cell were observed with different concentration of LHFM and citrulline respectively. Results suggested that, under the condition with 4 mM citrulline. Higher percentage of LHFM increased the number of IPEC-J2 cells; 3%LHFM_Cit-4mM exhibited weaker effects when compared to 4%LHFM_Cit-4mM and 5%LHFM_Cit-4mM (both P < 0.05). Similarly, increased concentration of citrulline showed stronger effect when cell numbers under 5%LHFM_Cit-2mM and 5%LHFM_Cit-4mM (P < 0.05) were considered. Results suggested that 5%FM with LH511 and 4 mM citrulline significantly stimulated the cell growth of IPEC-J2 cells.

### Adhesion of *Lactobacillus helveticus* ASCC 511 and anti-adhesion effect of enriched citrulline fermented milk with *Lactobacillus helveticus* ASCC 511 against pathogenic *Escherichia coli* in IPEC-J2 cells

**Figure 2:**
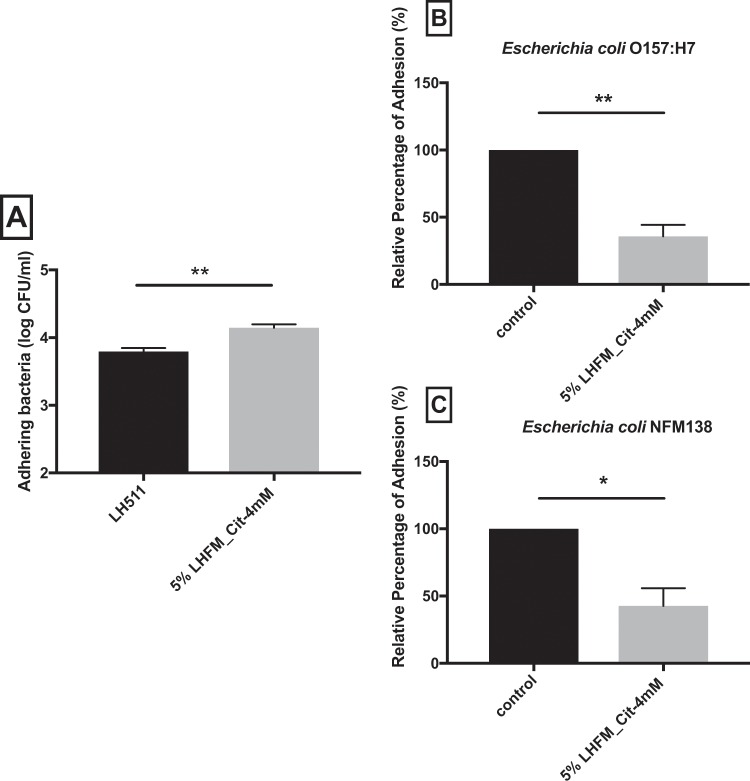
Adhesion assay. (**A**) Adhesion level of *L. helveticus* ASCC 511 co-incubated with enriched citrulline fermented milk in IPEC-J2 cell lines. Enriched citrulline fermented milk with *L. helveticus* ASCC 511 inhibited the adhesion of different type of *Escherichia coli*: (**B**) *Escherichia coli* PELI0480 (O157:H7) and (**C**) *Escherichia coli* NFM138 on IPEC-J2 cells. Results are represented as mean ± SEM, n = 3. Significance shown as *P < 0.05 and **P < 0.01, determined by t-test.

Adhesion of probiotics on intestinal epithelial cells is believed to effectively inhibit the attachment of pathogens. The number of adhered LH511 in IPEC-J2 cells was increased from 3.8 ± 0.081 log CFU/ml to 4.15 ± 0.086 log CFU/ml when incubated with 5% (v/v) FM with live LH511 and 4 mM citrulline (P < 0.01) (Fig. 2A). To further elucidate the effects of LH511 on bacterial adhesion in IPEC-J2 cells, the adhesion level of two pathogenic *E. coli* (O157:H7 and NFM138) in IPEC-J2 cells incubated with 5%LHFM_Cit-4mM for 2 h was examined. Figure 2B,C suggest that the adhesion level of O157:H7 and NFM138 was decreased to 35.79% ± 14.77 and 42.74% ± 22.58 when incubated with 5%LHFM_4mM when compared with the control (both P < 0.05).

### Citrulline-enriched fermented milk with *Lactobacillus helveticus* ASCC 511 improved LPS-induced reduction in transepithelial electrical resistance (TEER) on IPEC-J2 cells

**Figure 3:**
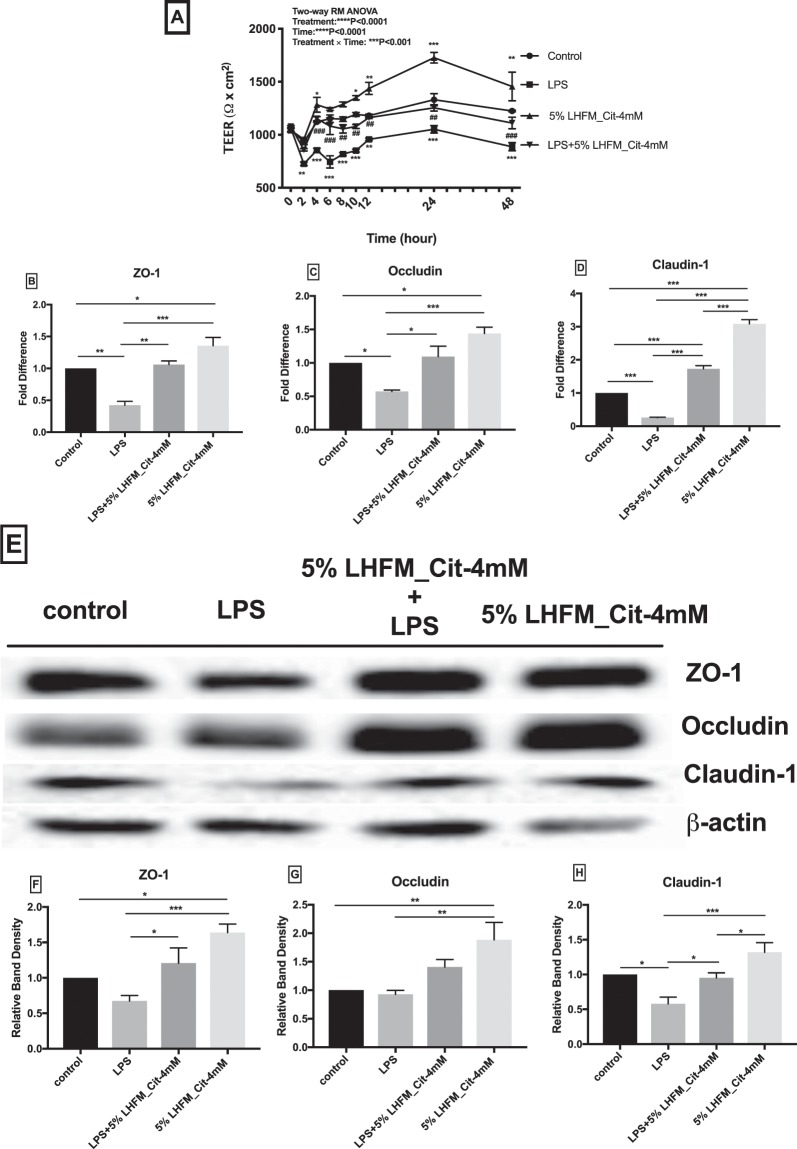
Effects of enriched citrulline fermented milk with *L. helveticus* ASCC 511 on IPEC-J2 cell integrity. (**A**) Transepithelial electrical resistance (TEER) in IPEC-J2 cells treated with lipopolysaccharides (LPS) and enriched citrulline fermented milk with *L. helveticus* ASCC 511 for 48 h (n = 3). Results are represented as mean ± SEM. Significance shown for the time × treatment as **P < 0.001 by Two-way RM ANOVA and *P < 0.05, **P < 0.01 and ***P < 0.001 as the difference compared with control and ^##^P < 0.01 and ^###^P < 0.001 as the difference of LPS + 5% LHFM_Cit-4mM compared with LPS at the same time point by Tukey’s multiple comparisons test. (**B**–**D**) mRNA expression of tight junction proteins (n = 3): (**B**) Zonula occluden-1 (ZO-1), (**C**) Occludin and (D) Claudin-1 in IPEC-J2 cells after incubated with LPS and enriched citrulline fermented milk with *L. helveticus* ASCC 511 after 24 h. (**E**) Representative charts of Western blot results; and densitometric analysis was performed and normalized to β-actin (n = 6): (F) ZO-1; (**G**) occludin; (**H**) Claudin. Results are represented as mean ± SEM. Significance shown for the difference compared between treatments as **P < 0.001 by One-way ANOVA and *P < 0.05, **P < 0.001 and ***P < 0.0001 as the difference compared between groups by Tukey’s multiple comparisons test.

To examine the protective effects of LHFM with citrulline in intestinal epithelial cells, TEER values were measured at 0, 2, 4, 6, 8, 10, 12, 24 and 48 h after being treated with LPS from *E. coli* O55:B5 (Fig. 3A). TEER of IPEC-J2 cells treated with LPS was significantly reduced from 2 h to 48 h as compared with the control from (all P < 0.01). At 2, 6 and 8 h, 5%LHFM_Cit-4mM had no significant effect on TEER of IPEC-J2 cells compared with the control (all P > 0.05), but it showed a significant stimulation during 10 h to 48 h (all P < 0.05). Overall, TEER of IPEC-J2 cells was enhanced from 14.3% to 29.8% when treated with 5%LHFM_Cit-4mM for 48 h. Results suggested that treatment with 5%LHFM_Cit-4mM regulated the LPS-induced decline on TEER, it showed a significant greater value than LPS group during 4 h to 48 h (all P < 0.05) and returned to normal values that had no significant difference compared with the control (all P > 0.05).

### Citrulline-enriched fermented milk with *Lactobacillus helveticus* ASCC 511 regulated LPS-induced TJ mRNA and protein expression and distribution of TJ structure in IPEC-J2 cells

**Figure 4:**
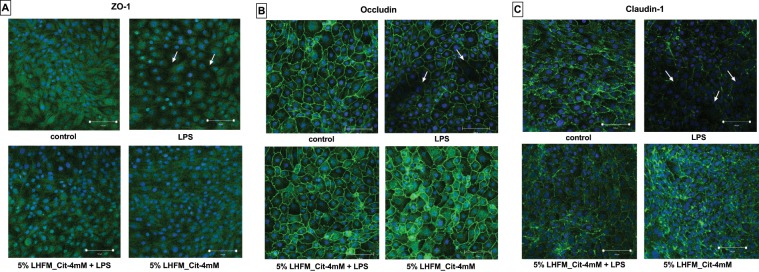
Effects of enriched citrulline fermented milk with *L. helveticus* ASCC 511 on distribution of tight junction proteins (**A**) ZO-1; (**B**) Occludin and; (**C**) Claudin-1 on IPEC-J2 cell. The cell monolayer was stained for the ZO-1, Occludin and Claudin-1 (green) and the nucleus (blue). The intensity of the tight junction proteins was decreased in LPS-infected cells compared to control cells and the tight junction belts were disturbed were present (arrows).

The protective effects of 5%LHFM_Cit-4mM on intestinal epithelial integrity were investigated by examining its effects on TJ proteins. We determined the mRNA expression of ZO-1, occludin and claudin-1 after being treated with 5%LHFM_Cit-4mM and LPS after 24 h (Fig. 3B–D). When incubated with LPS, 5%LHFM_Cit-4mM down-regulated the RNA expression of ZO-1, occludin and claudin-1 compared with the control (all P < 0.05) that reduced to 0.42 ± 0.11-, 0.57 ± 0.04- and 0.26 ± 0.02-fold, respectively. Treatment with 5%LHFM_Cit-4mM had a significant enhancing effect on RNA expression of ZO-1 with 1.36 ± 0.23-fold increase, occludin with 1.44 ± 0.17-fold increase, claudin-1 with 3.09 ± 0.22-fold increase (all P < 0.05). When co-incubated with 5%LHRM_Cit-4Mm, *L. helveticus* enriched with citrulline significantly attenuated the LPS-induced decline of mRNA expression of all TJ proteins (all P < 0.05), mRNA expression level of ZO-1 and occludin were regulated to normal values that had no significant difference compared with the control (both P > 0.05), whereas claudin-1 expression level was significantly higher than the control (P < 0.05). Conversely, we used western blotting to examine the protein level of TJ proteins (Fig. 3F–H). It showed similar results as with mRNA expression of these TJ proteins. However, LPS was reduced in all TJ proteins level, however, it was only statistically significant in claduin-1 (P < 0.05). It also showed the same order of reduction level: claudin-1 (0.58 ± 0.24) > ZO-1 (0.64 ± 0.16) > occludin (0.93 ± 0.16), when compared with the mRNA expression of these proteins. There was a significant stimulation in the protein levels of ZO-1 and occludin after 5%LHRM_Cit-4mM treatment as compared with the control (both P < 0.01), except in claudin-1. Administration of 5%LHRM_Cit-4mM also restored all TJ proteins level reduced by LPS, except that it was not significant in occludin (P = 0.22). We also used confocal imaging to determine the effects of 5%LHFM_Cit-4mM on the distribution of TJ structure in IPEC-J2 cells (Fig. 4A–C). The results indicated the ZO-1, occludin and claudin-1 were presented the continuous and circumferential distribution in non-infected (control) IPEC-J2 cells. LPS infected cells showed a discontinuous distribution and fainter staining in all TJ proteins compared with the control cells. In contract, treatment of 5%LHFM_Cit-4mM exhibited a stronger and brighter staining in the cell membrane and cytoplasm. Meanwhile, co-incubated 5%LHFM_Cit-4mM attenuated the disordered structure induced by LPS and showed a protective effect against LPS damage by improving the TJ proteins distribution.

### Citrulline-enriched fermented milk with *Lactobacillus helveticus* ASCC 511 regulated LPS-induced toll-like receptors mRNA expression in IPEC-J2 cells

**Figure 5:**
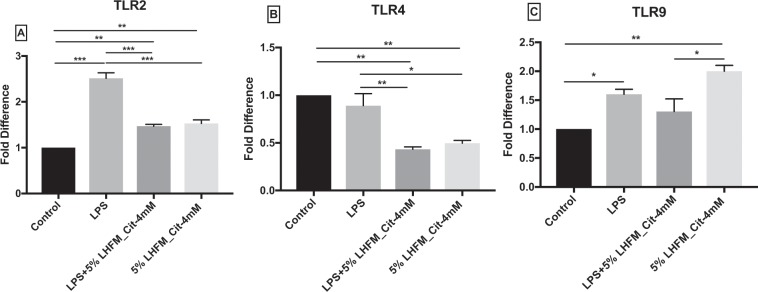
mRNA expression of toll-like receptors (TLR): (**A**) TLR2, (**B**) TLR4 and (**C**) TLR9 in IPEC-J2 cells after incubated with lipopolysaccharides (LPS) and enriched citrulline fermented milk with *L. helveticus* ASCC 511 after 24 h. Results are represented as mean ± SEM, n = 3. Significance shown for the difference compared between treatments as **P < 0.001 by One-way ANOVA and *P < 0.05, **P < 0.001 and ***P < 0.0001 as the difference compared between groups by Tukey’s multiple comparisons test.

To determine the effects of 5%LHFM_Cit-4mM on toll-like receptors (TLRs) on inflammatory response in LPS-activated cells, the mRNA expression of TLR2, TLR4 and TLR9 were selected in this study. Figure 5A–C suggested that the mRNA expression of TLR2 and TLR9 were increased by 2.51 ± 0.21-fold and 1.60 ± 0.15-fold when treated with LPS, whereas TLR4 exhibited no significant change (P = 0.66). The mRNA expression of TLR2 and TLR9 was significantly increased as compared with the control in 5%LHFM_Cit-4mM treatment with 1.53 ± 0.14 fold and 2.00 ± 0.17, respectively. However, when treated with 5%LHFM_Cit-4mM, fermented milk enriched with citrulline significantly lowered the mRNA expression of TLR4 to 0.50 ± 0.05 fold. Co-treatment of 5%LHFM_Cit-4mM with LPS showed a significant down-regulating effect on TLR2 and TLR4 on LPS-induced condition, but not on TLR9 (P = 0.40).

### Citrulline-enriched fermented milk with *Lactobacillus helveticus* ASCC 511 regulated LPS-induced negative regulators of toll-like receptors signalling pathway mRNA expression in IPEC-J2 cells

**Figure 6:**
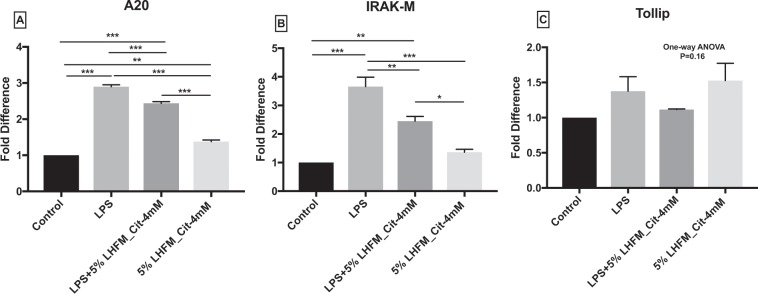
mRNA expression of negative regulators of toll-like receptors signaling pathway: (**A**) A20, (**B**) IRAK-M and (**C**) Tollip in IPEC-J2 cells after incubated with lipopolysaccharides (LPS) and enriched citrulline fermented milk with *L. helveticus* ASCC 511 after 24 h. Results are represented as mean ± SEM, n = 3. Significance shown for the difference compared between treatments as ***P < 0.0001 by One-way ANOVA and *P < 0.05, **P < 0.001 and ***P < 0.0001 as the difference compared between groups by Tukey’s multiple comparisons test.

The role of anti-inflammatory effects of 5%LHFM_Cit-4mM on LPS-induced IPEC-J2 cells was examined in terms of the expression of negative regulators of TLRs. The mRNA expression of A20, IRAK-M and Tollip after incubation with 5%LHFM_Cit-4mM and LPS is shown in Fig. 6A–C. The mRNA expression of A20 and IRAK-M was significantly higher in the LPS group as compared with the control with 2.90 ± 0.09- and 3.66 ± 0.57-fold increase, respectively. Treatment with 5%LHFM_Cit-4mM significantly increased the A20 expression by 1.38 ± 0.08 fold (P < 0.01), which also showed the stimulating effects on IRAK-M, although it was not statistically significant (P = 0.55). The 5%LHFM_Cit-4mM down regulated the mRNA expression when treated with LPS in the A20 and IRAK-M as compared with that treated with LPS-activated condition (both P < 0.01) and also showed higher expression as compared with the 5%LHFM_Cit-4mM group (both P < 0.05). Although the expression of Tollip was not significantly affected by all treatments (P = 0.16). There was only 1.38 ± 0.35- and 1.53 ± 0.42-fold increase in the LPS and 5%LHFM_Cit-4mM group, respectively.

### Citrulline-enriched fermented milk with *Lactobacillus helveticus* ASCC 511 down regulated LPS-induced inflammatory cytokines mRNA expression and concentration of IL-6 and IL-8 in IPEC-J2 cells

**Figure 7:**
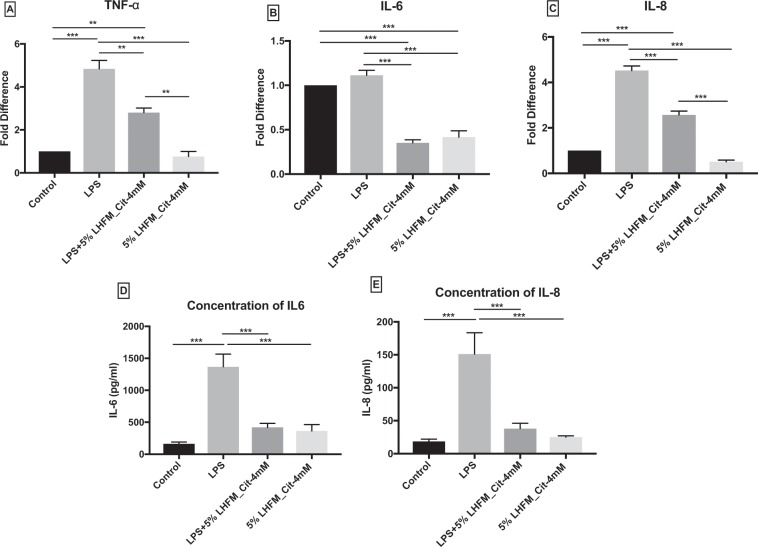
mRNA expression of inflammatory cytokines: (**A**) TNF-α, (**B**) IL-6 and (**C**) IL-8; and concentration of (**D**) IL-6 and (**E**) IL-8 in IPEC-J2 cells after incubated with lipopolysaccharides (LPS) and enriched citrulline fermented milk with *L. helveticus* ASCC 511 after 24 h. Results are represented as mean ± SEM; n = 3 (**A**–**C**), n = 6. (**D**,**E**) Significance shown for the difference compared between treatments as **P < 0.001 by One-way ANOVA and *P < 0.05, **P < 0.001 and ***P < 0.0001 as the difference compared between groups by Tukey’s multiple comparisons test.

The effects of 5%LHFM_Cit-4mM on the mRNA expression of inflammatory cytokines in IPEC-J2 cells, particularly TNF-α, IL-6 and IL-8 were investigated in this study (Fig. 7A–C) and concentrations of IL-6 and IL-8 were detected by the ELISA method (Fig. 7D,E). After 24 h incubation, LPS was significantly increased the TNF-α and IL-8 expression by 4.8 ± 0.68- and 5.2 ± 0.34-fold (both P < 0.05); however, there was no significant increase in the IL-6 expression (P = 0.89). Meanwhile, 5%LHFM_Cit-4mM significantly reduced the mRNA expression of IL-6 by 0.42 ± 0.12 fold (P < 0.001) and it showed 0.76 ± 0.41 and 0.51 ± 0.12-fold reduction in the TNF-α and IL-8 levels, though there were no statistical differences (P = 0.90 and P = 0.12, respectively). Treatment with 5%LHFM_Cit-4mM improved all three inflammatory cytokines mRNA expression stimulated by LPS (all P < 0.01), whereas IL-6 was only 0.35 ± 0.06-fold increase, that was at similar expression level as with 5%LHFM_Cit-4mM treatment (P = 0.86) and significantly lower than the control (P < 0.001). The concentration of IL-6 and IL-8 in LPS was significantly increased from 163.83 ± 67 to 1367.31 ± 486 and from 18.55 ± 8.52 to 159.10 ± 79 pg/ml, respectively, as compared with the control (both P < 0.001). Treatment with 5%LHFM_Cit-4mM showed a significant lower concentration of IL-6 and IL-8 compared with LPS (both P < 0.001), however, there were no lowering effects on both cytokines compared with the control (P = 0.62 and P = 0.99, respectively). While 5%LHFM_Cit-4mM greatly decreased both the cytokines concentration to 421.58 ± 150 and 38.08 ± 19 pg/ml, repectively, which was increased by LPS.

### Citrulline-enriched fermented milk with *Lactobacillus helveticus* ASCC 511 regulated LPS-induced early apoptosis in IPEC-J2 cells

**Figure 8:**
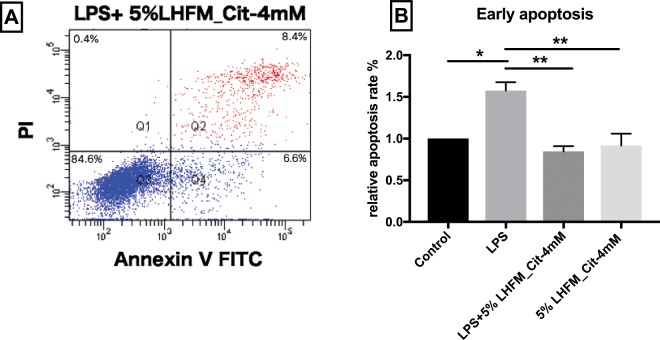
The flow cytometry dot plots of the effect of *L. helveticus* ASCC 511 fermented milk with citrulline on early apoptosis in IPEC-J2 cells (**A**) and the analysis of early apoptosis change. (**B**) Results are represented as mean ± SEM, n = 3. Significance shown for the difference compared with control as *P < 0.05 and **P < 0.01 by One-way ANOVA.

The anti-apoptotic effects of 5%LHFM_Cit-4mM were investigated by flow cytometry (Fig. 8A,B). Early apoptosis of IPEC-J2 cells was significantly increased by 1.6 ± 0.17-fold when exposed to LPS (P < 0.05). Although treatment with 5%LHFM_Cit-4mM did not effectively reduce the early apoptosis as compared with the control (P = 0.92). Co-incubation with LPS significantly regulated the LPS-induced higher apoptosis condition back to the normal level (P < 0.01).

## Discussion

In this study, we examined the effects of citrulline enriched FM with live LH511 on the integrity, functions and inflammatory responses against *E. coli* infection of intestinal epithelium by using IPEC-J2 cells. IPEC-J2 cell line is a non-transformed intestinal columnar epithelial cell line derived from small intestine tissue (mid-jejunum) from neonatal piglet^30^ and has been widely applied in many studies. IPEC-J2 cell line has been determined as a suitable and reliable *in vitro* model for the intestinal epithelium^31^. IPEC-J2 cells has higher similarity with human physiology than other mammal cell lines, and it has been proved showing similar result with other human cell lines^30^.

We first investigated the effects of FM, FM with live LH511 (LHFM) and LHFM with citrulline on the growth of intestinal epithelium cells. We found that 1–5% (v/v) FM, LH511 alone and 1–5% (v/v) LHFM show a trend of increase in the relative cell number of IPEC-J2 cells. Generally, FM was found to exhibit cell growth-promoting effect on IPEC-J2 cells and greater effect when added with live LH511 on a dose-dependent manner on addition with citrulline. Interestingly, some results showed that the relative cell numbers were slightly decreased when LHFM was combined with 2 mM or 4 mM citrulline, compared to LHFM alone. For example, the relative cell number of 1%LHFM was higher than control, 1%LHFM_Cit-2mM and 1%LHFM_Cit-4mM. However, 4%LHFM showed higher relative cell number than control but lower than 4%LHFM_Cit-2mM and 4%LHFM_Cit-4mM. These results suggested that LHFM alone might also have the cell growth promoting effect on IPEC-J2 cells, and the synergistic effect of combination of LHFM with citrulline could have contributed when combined with an optimal dosage of FM. The cell growth-promoting effects were predominantly increased when incubated with 5% (v/v) FM with live LH511 and 4 mM citrulline (5%LHFM_Cit-4mM) compared with the control and other combination groups (All P < 0.05) (Fig. 1), thus this combination is considered as the optimal dosage and therefore used in the following assays. These trophic effects of probiotics fermented milk and citrulline were supported by similar results from previous studies. A recent *in vitro* study by Chen, *et al*.^20^ reported that 50 ml/L FM containing 3 × 10^8^ CFU/ml of live *L. paracasei* 01 in the culture medium had a significant effect as compared with the control and had higher effect than 10 ml/L FM with heat-killed *L. paracasei* 01 in Caco-2 cells. Stadelmann, *et al*.^32^ reported that 0.4 mM citrulline improved growth of TC7 cells. Meanwhile, other *in vivo* studies suggested that oral supplementation of *L*. *casei* DN-114 001 FM and citrulline could stimulate growth in mucosal weights and villous area of small intestine of mice^33^ and piglets^34^. The cell growth-promoting effects of *L. helveticus* fermented milk on intestinal epithelial cells could be due to increased DNA synthesis and mitochondrial dehydrogenase activities^19^.

*L. helveticus* strain has been found adhering to human intestinal epithelial cells *in vitro* more than *L. acidophilus* and *L. plantarum* strain do^24^. We tested the adhesion capacity of LH511 in this study and found that the adhesion ability LH511 to IPEC-J2 was improved on incubation with FM with live LH511 and citrulline. Probiotics have been observed capable of protecting intestinal epithelium against pathogenic infections; this might be associated with their adhesive ability^35^. In this study, we observed that 5%LHFM_Cit-4mM effectively reduced the adhesion of both EHEC (O157:H7) and EIEC (NFM138) to IPEC-J2 cells. KS300, another strain of *L. helveticus*, was able to inhibit the growth and adhesion of uropathogenic *E. coli* IH11128 and *Gardnerella vaginalis* in HeLa cells^36^. It is suggested that these inhibitory effects of *L. helveticus* are due to the presence of their surface-layer proteins (Slps) located outside the outer cell wall in a paracrystalline layer. It has shown a significant inhibitory effect on the adhesion of O157: H7 to intestinal epithelial cells^37^. Moreover, anti-adhesion ability of probiotics FM was also described by an *in vitro* study of Zhao and Shah^38^, in which the milk was fermented with *Streptococcus thermophilus* ASCC 1275, *L. plantarum* ASCC 276 and *L. plantarum* ASCC 292. The product was shown to reduce the adhesion of *E. coli* O157:H7 PELI 0480, *Cronobacter sakazakii* ATCC 29544, *Staphylococcus aureus* CMCC 26003 and *Listeria monocytogenes* CMCC 54001 in Caco-2 cells. Apart from the anti-adhesion effect, the positive functions of milk fermented by *L. helveticus* against pathogenic infections have been reported in previous *in vivo* studies by LeBlanc, *et al*.^39^ and Tellez, *et al*.^40^. They demonstrated that peptide fraction derived from *L. helveticus-*FM supernatants protected BALB/c mice against *E. coli* O157:H7 and *Salmonella enteritidis* infection by enhancing the total immunoglobulin A (IgA)-secreting B lymphocytes in the intestinal lamina propria and affected the virulence gene expression.

Pathogenic infection impairs the intestinal epithelium integrity by altering the epithelial permeability and disturbing the epithelial structure, leading to uncontrolled entry of solutes and pathogens. Our findings showed that 5%LHFM_Cit-4mM could improve the intestinal epithelium integrity in normal condition and restored the barrier disruption of intestinal epithelium induced by *E. coli* LPS (Fig. 3A). Different strains of probiotics have been widely investigated for the intestinal barrier protecting effects, for examples, *L. plantarum*^10^*, L. acidophilus*^41^ and *L. reuteri*^42^, which have all been reported to strengthen TEER in normal intestinal epithelium, whereas *L. plantarum*^12^, *L. casei* subsp. *rhamnosus*^43^, *L. rhamnosus* GG, *L. paracasei* and *L. Johnsonii*^44^ could ameliorate the TEER after pathogenic LPS-induced damage. Similar to our results, the intestinal protective effects of probiotics FM and *L. helveticus* was demonstrated by other studies. They reported that the administration of 5%FM with live probiotics and Slps extract from *L. helveticus* enhanced and preserved the TEER on normal^20^ and *E. coli* O157:H7-infected intestinal epithelium^37^.

The maintenance of TEER of intestinal epithelium is associated with the TJ proteins, such as occludin and ZO-1^10^. In this study, the increased TEER on 5%LHFM_Cit-4mM was related to the stimulated mRNA expression (Fig. 3B,C) and protein level of ZO-1 and occludin (Fig. 3F,G) and distribution of all TJ proteins (Fig. 4A–C). LPS infection weakened the TJ integrity by reducing TEER and mRNA expression and disordering the structure of TJ proteins. Interestingly, our results suggested that there was no significant reduction in ZO-1 and occludin protein levels in LPS-infected cells (P = 0.18 and P = 0.99) and observed only in claudin-1 (P < 0.05). With reference to the mRNA expression, our study suggested that mRNA expression of claudin-1 was lower than ZO-1 and occludin. This might imply that the protein transcription level of TJ proteins had been reduced but the protein level still remained at a high level at 24 h. This might explain that the mRNA expression level of ZO-1 and occludin was significantly decreased but the protein levels were non-significantly reduced. Additionally, the results of the confocal imaging were supported this view, the distribution of claudin-1 in LPS infection was observed to have the stronger discontinuous distribution and fainter staining than ZO-1 and occludin. Zhang, *et al*.^45^ also reported that occludin protein level did not significantly decline after enterotoxigenic *E. coli* (ETEC) infection. Subsequently, after co-incubation the 5%LHFM_Cit-4mM attenuated the cell integrity in LPS-infected cells by restoring TEER and regulated TJ proteins expression and distribution. Previous findings have shown that the significant enhancement of TJ proteins expression and TEER were improved by *Lactobacillus* strains regardless of whether in non-pathogenic infected cells or LPS-infected cells^9,10,42,44^. In addition, *Bifidobacterium*^46^ and *Lactobacillus* strain^47^ have been reported to ameliorate the disordered structure of TJ and protein level of TJ damaged by pathogenic infection. Reinforcement and protection of TJs against damage by probiotics has been also suggested to be modulated via TLRs signalling in intestinal epithelial cells^2^. In our study, TLR2 mRNA expression level was significantly stimulated by 5%LHFM_Cit-4mM as compared with the control, and it was associated with the increased protein expression of TJs. At the same time, 5%LHFM_Cit-4mM modulated the TLR2 expression and attenuated the damage on TJ integrity induced by LPS. The enhancing and protective effect of probiotic on TJ functions is suggested to act as the TLR2 ligands to activate TLR2 expression. TLR2 activation has an important role in enhancing TJ integrity via myeloid differentiation primary response protein (MyD88)-dependent phosphatidyl inositol 3-kinase (PI3K)/Akt pathway and reducing the peptidoglycan stimulation^48–50^. For example, treatment or pre-treatment of *L. rhamnosus* stimulated ZO-1 and occludin expression under normal and ETEC-infected condition that was regulated by modulation of TLR2 expression via the Akt pathway^45^; *L. casei* was found to activate ZO-1 protein level while TLR2 and pAkt expression was increased^51^.

Activation of TLR4 expression is a primary response to pathogen-associated molecular patterns (PAMPs) on Gram-negative bacteria; this induces the recruitment of MyD88 to activate the nuclear factor-κB (NF-κB) and mitogen activated protein kinases (MAPK) pathways regulated by IRAK-1 and TRAF-6 resulting in the production of pro-inflammatory cytokines^2^. LPS is a common TLR4 ligand, it destroys TJ integrity via activating TLR4 expression to induce dephosphorylation of threonine residues and phosphorylation of tyrosine residues of occludin^50,52^. LPS infection induces TLR4 expression to disturb the TJ integrity and elicits inflammatory response. Interestingly, our results showed that TLR4 expression level was not significantly stimulated with LPS, whereas TLR2 expression was increased. A similar result was also reported by Chen, *et al*.^53^. These results might be caused by the up-regulated TLR4 activated by LPS, which stimulates the pro-inflammatory mediators and activates TLR2 expression and then down-regulates its own expression^13,54^. Additionally, the up-regulated TLR2 induced by LPS is not able to enhance TJ functions due to non-stimulating effects on PI3/Akt pathway^55^. Probiotics have been demonstrated to modulate the over-expression of TLR2 and TLR4 induced by pathogenic infection in both *in vitro*^56^ and *in vivo* studies^57^. For example, Gao, *et al*.^52^ demonstrated that *L. rhamnosus* GG activated TLR2 and TLR9 expression and down-regulated the TLR2 and TLR4 expression stimulated by LPS on IPEC-J2 cells. Our findings are in consistent with their results; 5%LHFM_Cit-4mM activated TLR2 expression and decreased TLR4 expression as compared with the control, and co-incubated with 5%LHFM_Cit-4mM also showed to regulate the activated TLR2 and reduced TLR4 expression induced by LPS. Our findings imply that 5%LHFM_Cit-4mM might act as a TLR2 ligand to up-regulate the expression of TLR2 and contribute to the suppressive effect on TLR4, and also reinforce and protect TJs against LPS damage.

Apart from the ability of enhancement of TJ strength, the regulation ability of probiotics on TLR2 expression also plays an important role in the modulation of pro-inflammatory cytokines via induction of endogenous negative regulators of TLR signaling^2^. The protective effect of probiotics on TJs is due to its role as a TLR2 ligand to stimulate negative regulators to suppress TLR4-mediated inflammatory responses. We found that 5%LHFM_Cit-4mM was able to induce all negative regulators mRNA expression in non-LPS treated cells, although IRAK-M and Tollip were not statistically significant (P = 0.55 and P = 0.17, respectively). When incubated with LPS, it resulted in activation of A20 and IRAK-M expression in this study that is consistent with previous findings^16^. However, other studies by Li, *et al*.^57^ and Finamore, *et al*.^58^ demonstrated that A20, Tollip and IRAK-M expressions were suppressed in ETEC infection. The different response of negative regulators expression under pathogenic infection might be affected by the dosage of LPS. High dosage of LPS (>10 ng/ml) induced TLR4 activation to cause a robust pro-inflammatory response through the stimulation of NF-κB pathway. It also simultaneously increased the level of negative regulators such as IRAK-M, PI3K, MKP-1 and RelB^59^. Thus, the induction of negative regulators level might be caused by the high dosage LPS used in our study (1 μg/ml). Moreover, *L. acidophilus*^57^ and *L. amylovorus*^58^ activated A20, IRAK-M and Tollip expression in normal condition and in ETEC infection in order to alleviate the inflammatory response, which was similar with our findings. Co-incubation of 5%LHFM_Cit-4mM with LPS also showed the higher expression in A20 and IRAK-M as compared with the control and 5%LHFM_Cit-4mM group and regulated the expression level in LPS challenge. The mechanisms of negative regulators on modulation of TLRs signalling in inflammatory responses have been demonstrated in our results. As expected, LPS increased the pro-inflammatory cytokines expression (TNF-α and IL-8) and concentration (IL-6 and IL-8) due to the disruption of TLRs signalling, while IL-6 expression did not significantly stimulate, that was consistent with the results of Farkas, *et al*.^60^. Treatment with 5%LHFM_Cit-4mM decreased IL-6 expression and slightly reduced TNF-α and IL-8 expression in non-LPS treated cells, and suppressed TNF-α, IL-6 and IL-8 expression and IL-6 and IL-8 concentration in the presence of LPS. It was suggesting that 5%LHFM_Cit-4mM supressed the expression and production of pro-inflammatory cytokines in LPS inflection. It is speculated to associate by regulating TLR4 expression via the modulation of A20 and IRAK-M. Several studies reported the positive effects of probiotic in anti-inflammatory response in pathogenic challenge via regulating TLRs signalling pathway and negative regulators^17,52,61,62^. A recent study by Kanmani and Kim^16^ reported that several types of lactic acid bacteria attenuated LPS induced negative regulators (A20, Tollip and SIGIRR) and pro-inflammatory cytokines (IL-6 and TNF-α) in HepG2 cells at 12 h, but not significantly altered the mRNA expression of TLR2 and TLR4. Additionally, *Lactobabillus* strains FM also found to suppress TNF-α and IL-6 expression in a LPS-infected murine model^63^.

We also investigated the effects of 5%LHFM_Cit-4mM in LPS-induced apoptosis in IPEC-J2 cells. We showed that 5%LHFM_Cit-4mM significantly declined LPS-caused early apoptosis (Fig. 7B). The anti-apoptotic effect against LPS infection of 5%LHFM_Cit-4mM is speculated to contribute by the TLR2 activating ability. It is supported by a previous study, which reported that the epithelial barrier dysfunction induced by pathogenic infection was positively linked with the higher epithelial apoptosis^45^. Their results showed that treatment with *L. rhamnosus* up-regulated TLR2 to stimulate the expression of ZO-1 via PI3K/Akt pathway. Regulating TJ integrity by TLR2 expression of probiotics might be the possible mechanism on suppressing excessive apoptosis induced by pathogenic infection. The other strains of *Lactobacillus*, *L. acidophilus*, was able to protect LPS induced apoptosis on human umbilical vein endothelial cells (HUVEC)^64^. The mechanism of anti-apoptotic effects of 5%LHFM_Cit-4mM is not well understood. Further study on the PI3K/Akt pathway medicated by 5%LHFM_Cit-4mM is required; however, it appears that 5%LHFM_Cit-4mM efficaciously prevented LPS-induced damage on intestinal barrier functions.

In conclusion, despite the effects of citrulline alone in intestinal barrier functions is not well understood and further studies are needed, the current results demonstrated that the combination of 5% FM with live LH511 and 4 mM citrulline had the positive effects on intestinal barrier functions. It promoted the growth of intestinal epithelial cells and the adhesion of LH511, protected IPEC-J2 cells from pathogenic *E. coli* (O157:H7 and NFM138) adhesion and enhanced TJ integrity by stimulating TJ proteins level, activating TLR2 and TLR9 and diminishing TLR4 expression to induce A20 expression resulting in suppression of IL-6 level in LPS untreated cells. Whereas administration of 5%LHFM_Cit-4mM ameliorated the intestinal mucosal barrier functions reduced by LPS through restoring TEER values and TJ proteins level, regulated the TLRs signalling pathway and negative regulators expression and reduced the pro-inflammatory cytokines to alleviate inflammatory responses and damages caused by LPS. Thus, it appears that 5%LHFM_Cit-4mM may work as a novel functional health supplement for improving intestinal barrier functions.

## Material and Methods

### Cell culture

The IPEC-J2 cell line was donated by Dr. Wai Hung Sit, from the Department of Biological Sciences, the University of Hong Kong. Cells were grown in Dulbecco’s Modified Eagle medium (DMEM)/F-12 (DMEM/F12; Sigma-Aldrich, St. Louis, Missouri, USA), supplemented with 5% fetal bovine serum (Thermo Fisher Scientific Inc., Waltham, Massachusetts, USA), 1% ITS solution with insulin, transferrin, selenium and ethanolamine (GIBCO^®^, Life Technologies, Madrid, Spain) and kept at 37 °C in a humidified 5% CO_2_ atmosphere. IPEC-J2 cells were seeded in different types of tissue plates (see below) with cells at 5 × 10^5^ cell/ml cell concentration to conduct different assays when the cells reached confluence.

### Bacterial strains and preparation

*Lactobacillus helveticus* ASCC 511 (LH511) was obtained from the Dairy Innovation Australia Limited (ASCC, Werribee, Victoria, Australia). It was stored at −80 °C in 20% (v/v) glycerol (Sigma-Aldrich, Munich, Germany) and activated in 10 ml of de Man, Rogosa and Sharpe (MRS) broth (BD, New Jersey, USA) at 37 °C for 24 h. The activated cell was centrifuged at 5,000 × g at 4 °C for 10 min and washed in phosphate buffered saline (PBS) and then re-suspended in DMEM/F-12 and obtained approximately 3 × 10^7^ CFU/ml of bacteria.

### Sample preparation

To prepare the fermented milk, 2% (v/v) of LH511 was inoculated into sterile reconstituted skimmed milk (RSM) (12%, w/v) and incubated at 37 °C for 24 h. Then, fermented milk was centrifuged at 10,000 × g at 4 °C for 30 min. After centrifugation, fermented milk supernatant (FM) was filtered using a 0.22μm membrane to remove the bacteria and was stored at −20 °C until use.

The samples used in this study including: FM (LH511-removed fermented milk supernatant), LHFM (fermented milk supernatant with live 3 × 10^7^ CFU/ml LH511) and citrulline (Sigma-Aldrich, Munich, Germany).

### MTT assay

The determination of the growth promoting effects of fermented milk, *Lactobacillus helveticus* ASCC 511 and citrulline on IPEC-J2 cell by MTT assay was as previously described by Zhao and Shah^38^ with some modifications. IPEC-J2 cells were seeded in 96-well tissue culture plates (NUNC, Thermo Fisher Scientific, Waltham, Massachusetts, USA) until confluence, and divided into groups as follows: control group (without any treatment), 1%, 2%, 3%, 4% and 5% (v/v) of FM group (fermented milk supernatant removed LH511), LH511 group (treated with live 3 × 10^7^ CFU/ml LH511 alone), 1–5% of LHFM group (1–5% (v/v) of FM and treated with live 3 × 10^7^ CFU/ml LH511), 1–5% of LHFM_Cit-2mM group (1–5% (v/v) of FM and treated with live 3 × 10^7^ CFU/ml LH511 and 2 mM citrulline) and 1–5% of LHFM_Cit-4mM group (1–5% (v/v) of FM and treated with live 3 × 10^7^ CFU/ml LH511 and 4 mM citrulline). After 24 h incubation, 10 μl of 3-(4, 5-dimethylthiazol-2-yl)-2,5-diphenyltetrazolium bromide (MTT) (5 mg/ml) was added into the well and incubated at 37 °C for 2 h. The medium was then discarded and 100 μl of dimethylsufoxide (DMSO) was added in well and incubated at 37 °C for 30 min. The absorbance was measured at 570 nm in an ELISA reader.

### Pathogens

*Escherichia coli* PELI0480 (O157:H7) and *Escherichia coli* NFM138 were obtained from Prof. Dr. Hua Wei, State Key Laboratory of Food Science and Technology, Nanchang University and Prof. Dr. Wei Chen, State Key Laboratory of Food Science and Technology, Jiangnan University. All pathogens were cultivated in LB broth at 37 °C for 18–20 h before the assays.

### Adhesion assay

IPEC-J2 cells were seeded in 12 well tissue plates until confluence. Cells were washed two times with PBS to remove the antibiotics in the medium. Wells were divided as three groups: control (without any treatment), LH511 (treated with 3 × 10^7^ CFU/ml of LH511) and 5%LHFM_Cit-4mM (treated with 5% (v/v) of FM with 4 mmol/l citrulline and live 3 × 10^7^ CFU/ml of LH511). Simultaneously, concentrations at 3 × 10^7^ CFU/ml of *E. coli* PELI0480 (O157:H7) and NFM138 were added in the wells. After 2 h incubation, the supernatant was discarded and the wells were washed with PBS and bacteria adhered cells were trypsinized by 0.25% of trypsin-EDTA solution (GIBCO®) and the viable bacteria attached to cell line were plated on LB or MRS agar plates. The percentage of adhesion was calculated by the following equation:

$${\rm{Relative}}\,{\rm{Percentage}}\,{\rm{of}}\,{\rm{Adhesion}}\,( \% )=({\rm{C}}{\rm{F}}{{\rm{U}}}_{{\rm{s}}{\rm{a}}{\rm{m}}{\rm{p}}{\rm{l}}{\rm{e}}}/{\rm{C}}{\rm{F}}{{\rm{U}}}_{{\rm{c}}{\rm{o}}{\rm{n}}{\rm{t}}{\rm{r}}{\rm{o}}{\rm{l}}})\times 100,$$

where CFU_sample_ was the number of bacteria adhered in sample and CFU_control_ was the number of bacteria adhered in the control.

### Transepithelial electrical resistance assay (TEER)

To determine the effect of *Lactobacillus helveticus* ASCC 511 fermented milk with citrulline on intestinal epithelial cell integrity under LPS-induced damage, IPEC-J2 cells were seeded in a permeable 12 mm Transwell with 0.4μm pore polyester membrane inserts (Corning, USA) until confluence, and divided into four groups: control group (without any treatment), Lipopolysaccharide (LPS) from *Escherichia coli* O55:B5 (Sigma-Aldrich, Munich, Germany) (treated with 1 μg/ml LPS), LPS + 5%LHFM_Cit-4mM (treated with with 1 μg/ml LPS and 5% (v/v) of FM with 4 mmol/l citrulline and live 3 × 10^7^ CFU/ml of LH511) and 5%LHFM_Cit-4mM (treated with 5% (v/v) of FM with 4 mmol/l citrulline and live 3 × 10^7^ CFU/ml of LH511). TEER was measured by using an ohmmeter (model EVOM, WPI Inc., Florida, USA) at 0, 2, 4, 6, 8, 10, 12, 24 and 48 hr.

### Real time PCR

Total RNA was isolated from the cells using TRIzol™ Reagent (Invitrogen Corporation, Carlsbad, California, USA) according to the manufacturer’s protocol. RNA quality and quantity were determined by a NanoDrop Spectrophotometer (Thermo Fisher Scientific Inc., Waltham, Massachusetts, USA). Extracted RNA sample was reverse-transcribed to complementary DNA (cDNA) using a PrimeScriptTM RT Master Mix kit (Takara, Japan). Real-time PCR was conduced using an Applied Biosystems 7500 Real-Time PCR System (Applied Biosystems®, California, USA) with SYBR Green PCR Master Mix (Takara, Japan). The PCR system consisted of 5 μl of SYBR Green qPCR Mix, 1 μl of cDNA, 0.2μmol of each primer, 0.2μmol reference dye and 3.4 μl of distilled water in a final volume of 20 μl. The primers are shown in Table 1. β-actin was used as a housekeeping gene for the PCR reaction.

**Table 1:** Primers used for real-time PCR.

Genes	Primers	Sequences (5′-3′)	Reference
β-actin	Forward	TGCGGGACATCAAGGAGAAG	Yang, *et al*.^42^
	Reverse	AGTTGAAGGTGGTCTCGTGG	
Claudin-1	Forward	GCAGCAGCTTCTTGCTTCTC	
	Reverse	CTGGCATTGACTGGGGTCAT	
Occludin	Forward	ATCAACAAAGGCAACTCT	
	Reverse	GCAGCAGCCATGTACTCT	
ZO-1	Forward	GAGTTTGATAGTGGCGTT	
	Reverse	GTGGGAGGATGCTGTTGT	
IL-6	Forward	TGGCTACTGCCTTCCCTACC	Collado-Romero, *et al*.^67^
	Reverse	CAGAGATTTTGCCGAGGATG	
IL-8	Forward	TTCGATGCCAGTGCATAAATA	
	Reverse	CTGTACAACCTTCTGCACCCA	
TNF-α	Forward	CCTCTTCTCCTTCCTCCTG	
	Reverse	CCTCGGCTTTGACATTGG	
TLR-2	Forward	TCACTTGTCTAACTTATCATCCTCTTG	
	Reverse	TCAGCGAAGGTGTCATTATTGC	
TLR-4	Forward	GCCATCGCTGCTAACATCATC	
	Reverse	CTCATACTCAAAGATACACCATCGG	
TLR-9	Forward	CACGACAGCCGAATAGCAC	
	Reverse	GGGAACAGGGAGCAGAGC	
IRAK-M	Forward	TGGAGCAGCCTTGAATCCTT	Li, *et al*.^57^
	Reverse	TGGATAACACGTTTGGGAATCTT	
Tollip	Forward	TACCGTGGGCCGTCTCA	
	Reverse	CCGTAGTTCTTCGCCAACTTG	
A20	Forward	CCTCCCTGGAAAGCCAGAA	
	Reverse	GTGCCACAAGCTTCCTCACTT	

### IL-6 and IL-8

The concentration of IL-6 and IL-8 in supernatants from IPEC-J2 cell cultures after 24 h incubated with LPS was measured by using commercial ELISA kit (Aviva Systems Biology, San Diego, California, USA) according to the manufacturer’s instructions.

### Western blot

The IPEC-J2 cells were seeded in 60 mm dish plates until confluence and divided into groups as follows: control (without any treatment), LPS (treated with 1 μg/ml of LPS), LPS + 5%LHFM_Cit-4mM (treated with with 1 μg/ml LPS and 5% (v/v) of FM with 4 mmol/l citrulline and live 3 × 10^7^ CFU/ml of LH511) and 5%LHFM_Cit-4mM (treated with 5% (v/v) of FM with 4 mmol/l citrulline and live 3 × 10^7^ CFU/ml of LH511). Cells were collected after 24 hr. The determination of the effect of *Lactobacillus helveticus* ASCC 511 fermented milk with citrulline on IPEC-J2 cell was as per Yang, *et al*.^42^ with some modifications. IPEC-J2 cells were collected and scraped by using radio-immunoprecipitation assay (RIPA) buffer (150 mM sodium chloride, 1% NP-40, 0.25% sodium deoxycholate, 0.1% SDS, 50 mM Tris-HCl at pH 7.4 and 1 mM EDTA) and then maintained constant agitation for 15 min at 4 °C. IPEC-J2 cells were centrifuged at 12,000 × g at 4 °C for 10 min and the supernatant was collected. Protein contents were determined using Bradford’s method. Samples with same protein concentrations were added in 6x loading buffer and heated in 100 °C for 5 min. Samples were subjected to 9% SDS-PAGE and transferred to polyvinylidene difluoride (PVDF) membranes at 100 V for 90 min. The membranes were blocked in 5% skim milk for 1 h and then incubated with primary antibodies against zonula occluden-1 (ZO-1) (Bioss Inc., Massachusetts, USA), claudin-1 (Cell Signaling Technology, Danvers, MA, USA), occludin (Abcam, Cambridge, United Kingdom) and β-actin (Cell Signaling Technology, Danvers, Massachusetts, USA) overnight at 4 °C. After overnight incubation, the membranes were washed by 1X TBST and incubated with horseradish peroxidase conjugated secondary antibodies (Sigma-Aldrich, Munich, Germany) for 1 h at room temperature. The membranes were washed by TBST and then analyzed by ChemiDoc^TM^ XRS + imaging system (Bio-Rad, Hercules, California, USA).

### Confocal immunofluorescence microscopy

The determination of the effect of *Lactobacillus helveticus* ASCC 511 fermented milk with citrulline on IPEC-J2 cell was as per Luo, *et al*.^65^ with some modifications. The IPEC-J2 cells were washed with PBS and fixed in 4% formaldehyde for 15 min at room temperature. After fixation, IPEC-J2 cells were permeabilized with PBS with Triton X-100 at room temperature and then blocked with goat serum for 1 h at room temperature. Cells were incubated with primary antibodies against zonula occluden-1 (ZO-1) (Bioss Inc., Massachusetts, USA), claudin-1 (Cell Signaling Technology, Danvers, Massachusetts, USA), occludin (Abcam, Cambridge, United Kingdom) at 4 °C for overnight. After washing with PBST, cells were incubated with FITC-conjugated specific secondary antibody (Sigma) at room temperature for 1.5 hr in the dark. Cells were stained with DAPI (4′,6-diamidino-2-phenylindole) for 5 min and then detected by confocal laser scanning microscopy (Carl Zeiss LSM 710 NLO).

### Flow cytometry

The anti-apoptosis effect of *Lactobacillus helveticus* ASCC 511 fermented milk with citrulline in IPEC-J2 cells was determined by flow cytometric method as previously described by Tang, *et al*.^66^ with some modifications. IPEC-J2 cells were seeded in 35 mm dish plates until confluence and divided into groups as follows: control (without any treatment), LPS (treated with 1 μg/ml of LPS), LPS + 5% LHFM_Cit-4mM (treated with with 1 μg/ml LPS and 5% (v/v) of FM with 4 mmol/l citrulline and live 3 × 10^7^ CFU/ml of LH511) and 5%LHFM_Cit-4mM (treated with 5% (v/v) of FM with 4 mmol/l citrulline and active 3 × 10^7^ CFU/ml of LH511). After 24 h incubation, the supernatant was discarded and the wells were washed with PBS and cells were trypsinized by 0.25% of trypsin-EDTA solution (GIBCO®, Life Technologies; Spain). About 1 × 10^6^ cells were collected and centrifuged at 1000 × g for 5 min. Cells were washed two times with ice-cold PBS and re-suspended in 1 ml of Annexin V binding buffer and then 100 μl of the cell solution were transferred to a 5 ml tube and 1 μl of FITC Annexin V and 1 μl of PI were added. After 15 min incubation at room temperature in the dark, 400 μl of binding buffer was added and then apoptotic cells were determined using flow cytometer (BD FACSCalibur, California USA).

### Statistical analysis

All data were analyzed using GraphPad Prism 7 Software (GraphPad Software, San Diego, CA, USA). One-way analysis of variance (ANOVA) was used with Tukey’s multiple comparisons test to determine the difference between all treatment groups on MTT, adhesion assay, qPCR, western blot and flow cytometry analysis. Results of adhesion of probiotic bacteria was determined by t-test. Two-way repeated measure ANOVA with Sidak’s multiple comparisons test to analyze the results of TEER. Results are expressed as mean ± SEM.

## Supplementary information

Supplementary information_SREP-19-03945.
