# Supplementary material for: The protective effects of enriched citrulline fermented milk with Lactobacillus helveticus on the intestinal epithelium integrity against Escherichia coli infection
Source: Sci Rep. 2020 Jan 16;10:499. doi: 10.1038/s41598-020-57478-w (PMC6965087; doi:10.1038/s41598-020-57478-w)
Supplement: Supplementary file 1 — Supplementary information_SREP-19-03945. [file 41598_2020_57478_MOESM1_ESM.pdf]

The protective effects of enriched citrulline fermented milk with *Lactobacillus helveticus* on the intestinal epithelium integrity against *Escherichia coli* infection

Sze Wing Ho, Hani El-Nezami and Nagendra P Shah*

Food and Nutritional Science, School of Biological Sciences, The University of Hong Kong, Pokfulam Road, Hong Kong

***Corresponding Author**

Nagendra P. Shah, Ph.D.

Professor of Food Science and Technology

Food and Nutritional Science; School of Biological Sciences

6N08, Kadoorie Biological Sciences Building;

The University of Hong Kong, Hong Kong

Tel:+852 2299 0836; Fax:+852 2559 9114; e-mail: npshah@hku.hk

Figure S1. Supplementary figures for ZO-1, occludin, claudin-1 and actin verified

by western blot:

Western blot analysis data for figure 1-10:

Figure 3E

Gel 1, 2 (ZO-1, occludin, claudin-1 and actin):

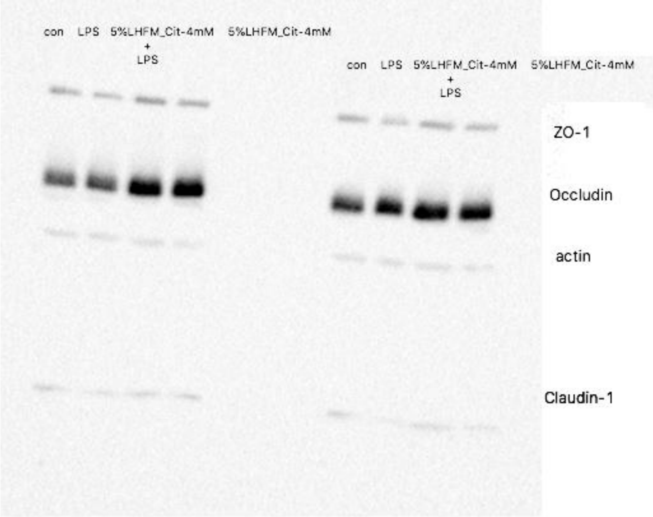

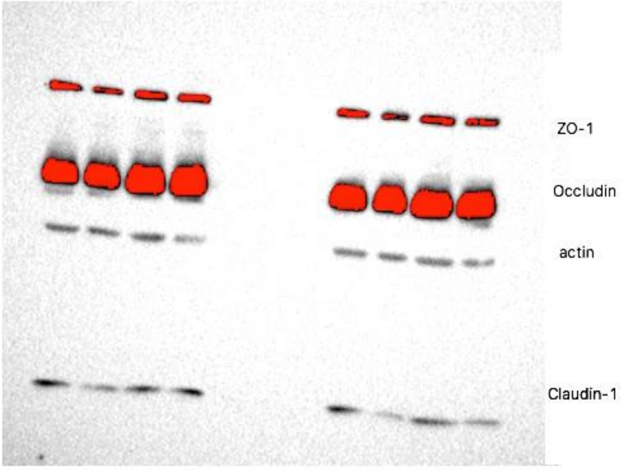

gel 3 (ZO-1, occludin, claudin-1 and actin):

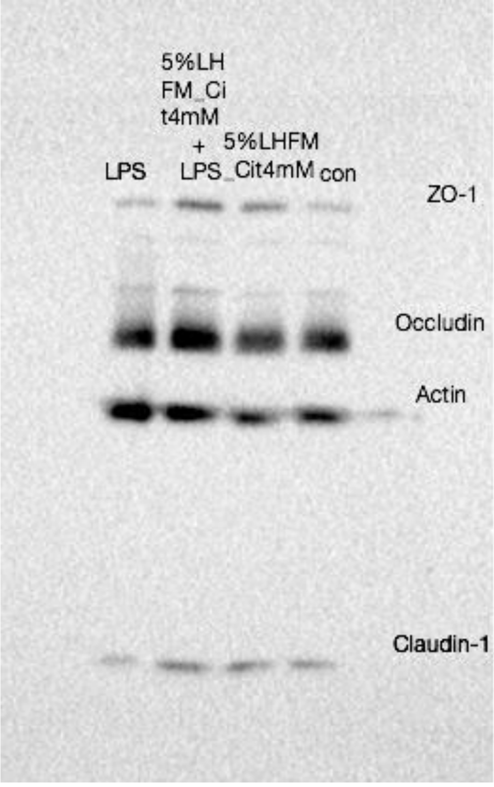

gel 4 (ZO-1 and Occludin):

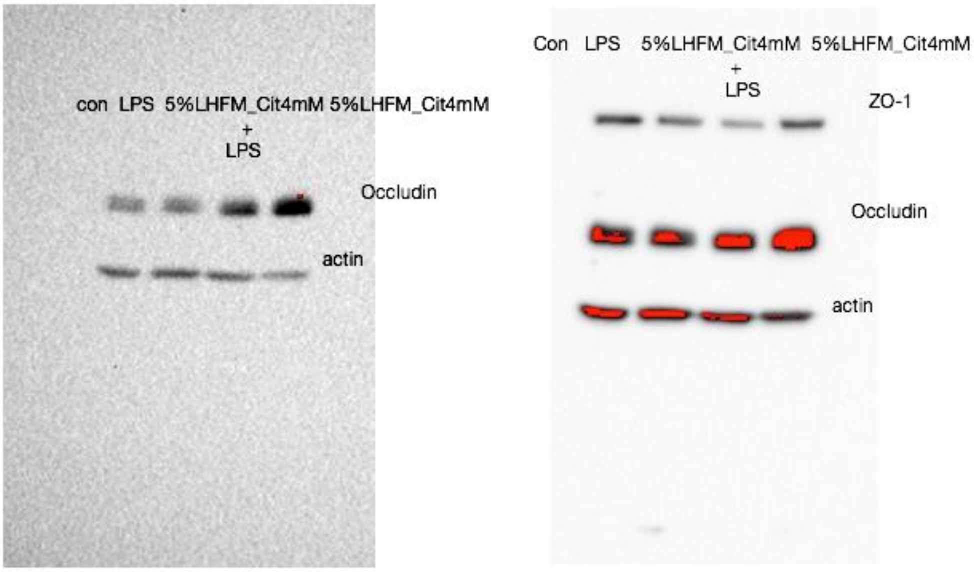

gel 5 (Occludin):

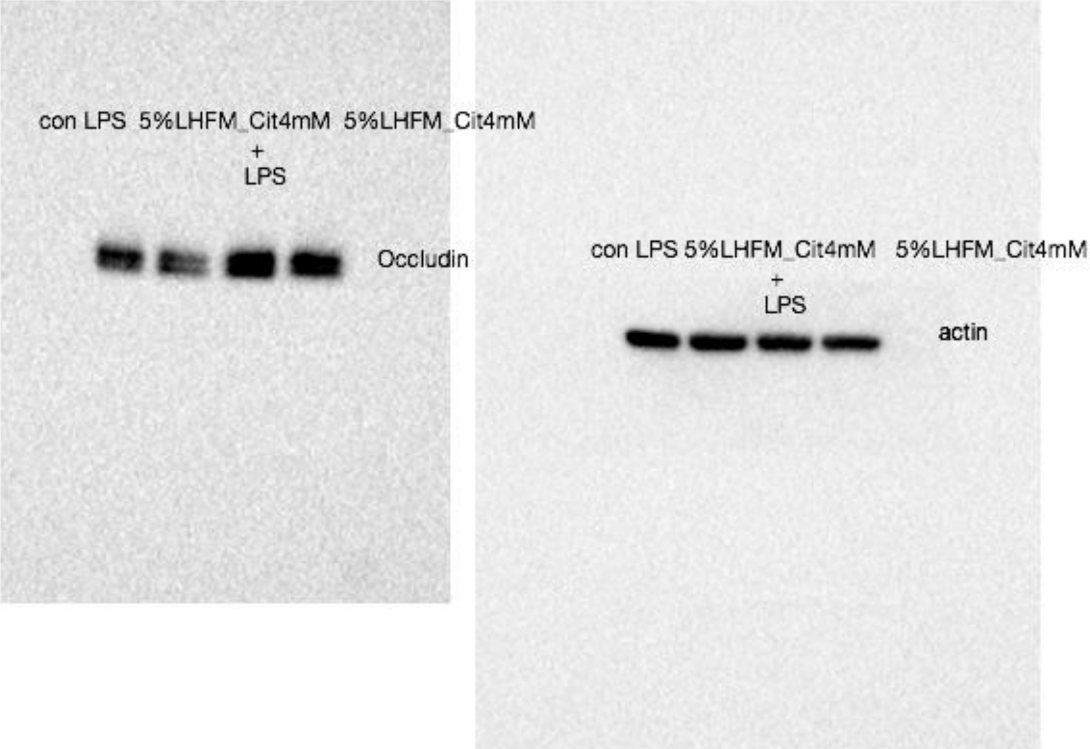

gel 5 (Occludin)

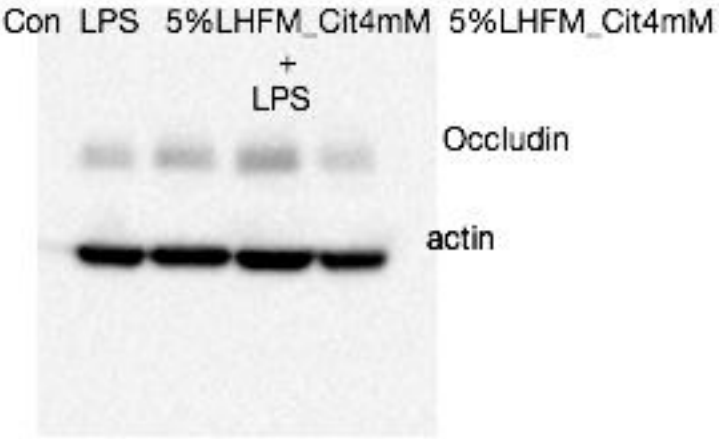

gel 6 (ZO-1):

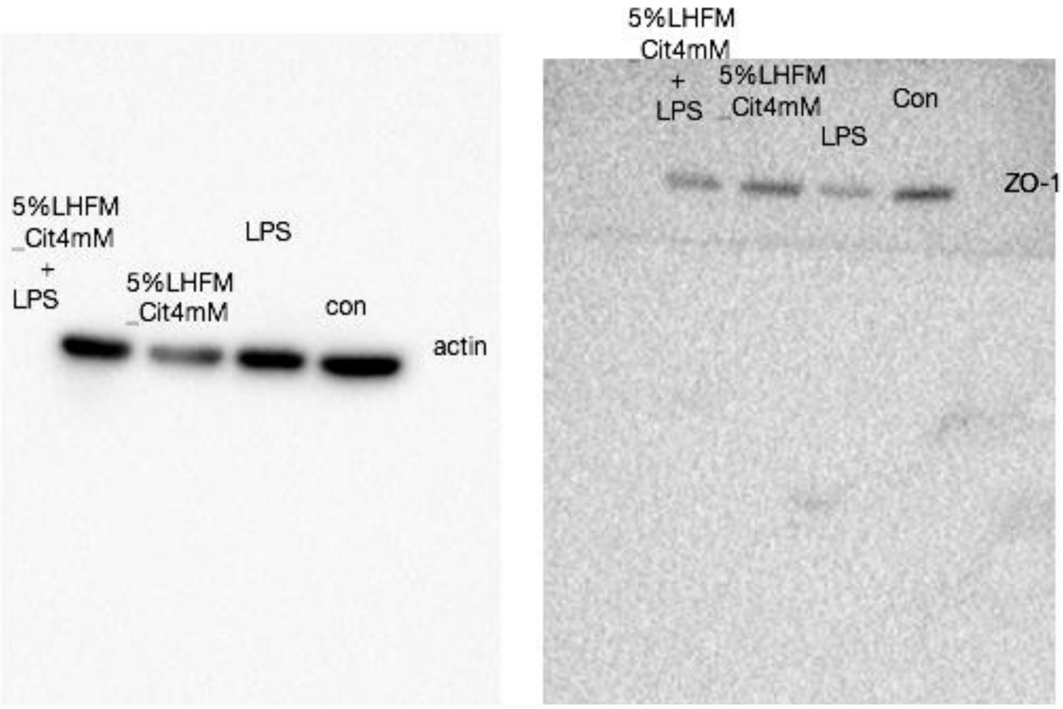

gel 7 (ZO-1

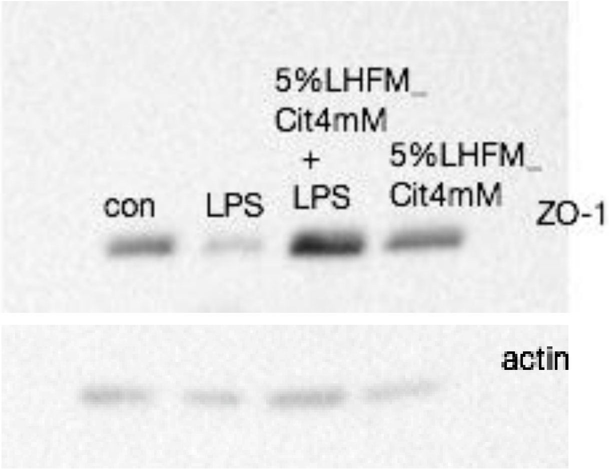

gel 8 (Claduin-1

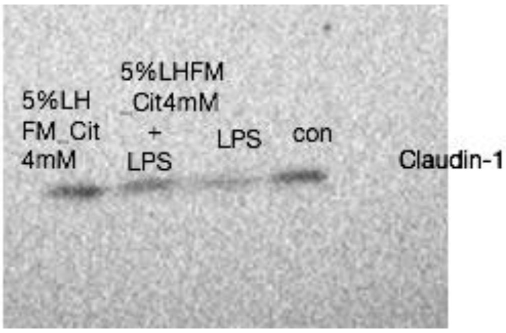

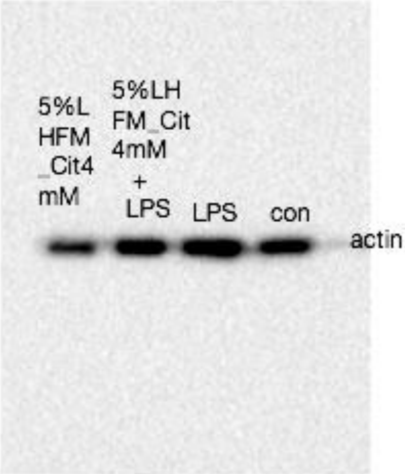

gel 9 (Claudin-1

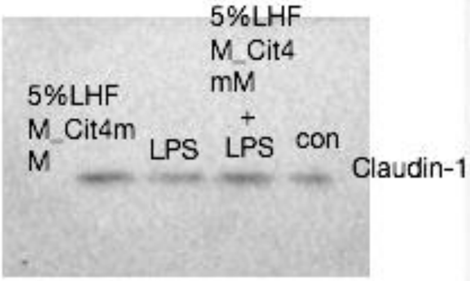

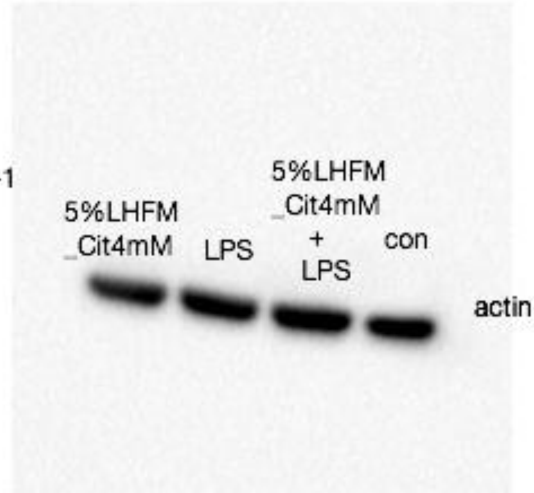

gel 10

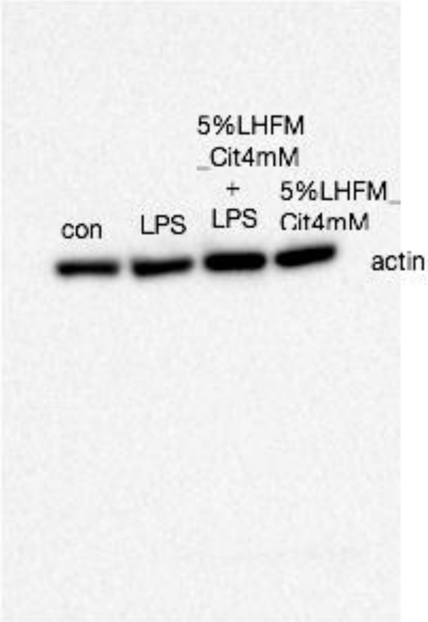

5%LHFM
_Cit4mM
+
LPS
con
LPS
5%LHFM_
Cit4mM
actin

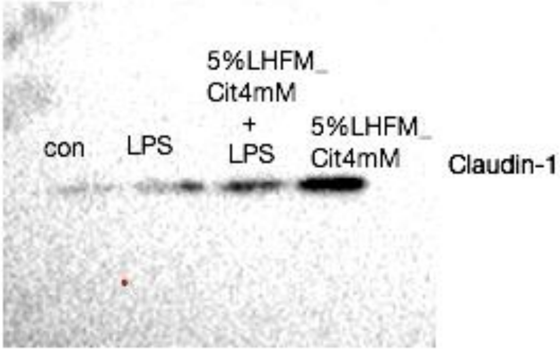

5%LHFM_
Cit4mM
+
LPS
con
LPS
5%LHFM_
Cit4mM
Claudin-1
